# Associative memory of phase-coded spatiotemporal patterns in leaky Integrate and Fire networks

**DOI:** 10.1007/s10827-012-0423-7

**Published:** 2012-10-04

**Authors:** Silvia Scarpetta, Ferdinando Giacco

**Affiliations:** 1 Department of Physics ‘E. R. Caianiello’, University of Salerno, 84084, Fisciano (SA), Italy; 2 INFN Unita’ di Napoli Gruppo coll. di Salerno, Fisciano, Italy; 3 Department of Environmental Sciences, Second University of Naples, 81100, Caserta, Italy

**Keywords:** Learning and memory, Phase-of-spikes coding, Storage capacity, Replay, Associative memory, Noise robustness, STDP

## Abstract

We study the collective dynamics of a Leaky Integrate and Fire network in which precise relative phase relationship of spikes among neurons are stored, as attractors of the dynamics, and selectively replayed at different time scales. Using an STDP-based learning process, we store in the connectivity several phase-coded spike patterns, and we find that, depending on the excitability of the network, different working regimes are possible, with transient or persistent replay activity induced by a brief signal. We introduce an order parameter to evaluate the similarity between stored and recalled phase-coded pattern, and measure the storage capacity. Modulation of spiking thresholds during replay changes the frequency of the collective oscillation or the number of spikes per cycle, keeping preserved the phases relationship. This allows a coding scheme in which phase, rate and frequency are dissociable. Robustness with respect to noise and heterogeneity of neurons parameters is studied, showing that, since dynamics is a retrieval process, neurons preserve stable precise phase relationship among units, keeping a unique frequency of oscillation, even in noisy conditions and with heterogeneity of internal parameters of the units.

## Introduction

It has been hypothesized that, in many areas of the brain, having different brain functionality, repeatable precise spatiotemporal patterns of spikes play a crucial role in coding and storage of information. Temporally structured replay of spatiotemporal patterns have been observed to occur during sleep, both in the cortex and hippocampus (Nadasdy et al. 1999; Ji and Wilson 2007; Euston et al. 2007; Lansink et al. 2009), and it has been hypothesized that this replay may subserve memory consolidation. The sequential reactivation of hippocampal place cells, corresponding to previously experienced behavioral trajectories, has been observed also in the awake state (awake replay) (Diba and Buzsaki 2007; Davidson et al. 2009; Girardeau and Zugaro 2011; Carr et al. 2011), namely during periods of relative immobility. Awake replay may reflect trajectories through either the current environment or previously, spatially remote, visited environments. A possible interpretation is that spatiotemporal patterns, stored in the plastic synaptic connections of hippocampus, are retrieved when a cue activates the emergence of a stored pattern, allowing these patterns to be replayed and then consolidated in distributed circuits beyond the hippocampus (Carr et al. 2011). Cross-correlogram analysis revealed that in prefrontal cortex the time scale of reactivation of firing patterns during post-behavioral sleep was compressed five- to eightfold relative to waking state (Euston et al. 2007; Schwindel and McNaughton 2011), a similar compression effect may also be seen in primary visual cortex (Ji and Wilson 2007). Internally generated spatiotemporal patterns have also been observed in the rat hippocampus during the delay period of a memory task, showing that the emergence of consistent pattern of activity may be a way to maintain important information during a delay in a task (Pastalkova et al. 2008).

Among repeating patterns of spikes a central role is played by phase-coded patterns (Siegel et al. 2009; Kayser et al. 2009; Montemurro et al. 2008), i.e. patterns with precise relative phases of the spikes of neurons participating to a collective oscillation, or precise phases of spikes relatively to the ongoing oscillation.

First experimental evidence of the importance of spike phases in neural coding was observed in experiments on theta phase precession in rat’s place cells (O’Keefe and Recce 1993; O’Keefe and Burgess 2005), showing that spike phase is correlated with rat’s position. Recently, the functional role of oscillations in the hippocampal-entorinal cortex circuit for path-integration has been deeply investigated (O’Keefe and Recce 1993; Lengyel et al. 2005a; O’Keefe and Burgess 2005; Euston et al. 2007; Geisler et al. 2007; McNaughton et al. 2006), showing that place cells and grid cells form a map in which precise phase relationship among units plays a central role. In particular it has been shown (Burgess et al. 2007; Blair et al. 2008; Welday et al. 2011) that both spatial tuning and phase-precession properties of place cells can arise when one has interference among oscillatory cells with precise phase relationship and velocity-modulated frequency.

Further evidence of phase coding comes from the experiments on spike-phase coding of natural stimuli in auditory and visual primary cortex (Montemurro et al. 2008; Kayser et al. 2009), and from experiments on short-term memory of multiple objects in prefrontal cortices of monkeys (Siegel et al. 2009).

These experimental works support the hypothesis that collective oscillations may underlie a phase dependent neural coding and an associative memory behavior which is able to recognize the phase coded patterns.

The importance of precise timing relationships among neurons, which may carry information to be stored, is supported also by the evidence that precise timing of few milliseconds is able to change the sign of synaptic plasticity (Markram et al. 2011). The dependence of synaptic modification on the precise timing and order of pre- and post-synaptic spiking has been demonstrated in a variety of neural circuits of different species. Many experiments show that a synapse can be potentiated or depressed depending on the precise relative timing of the pre- and post-synaptic spikes. This timing dependence of magnitude and sign of plasticity, observed in several types of cortical (Markram et al. 1997; Feldman 2000; Sjostrom et al. 2001) and hippocampal (Bi and Poo 1998; Magee and Johnston 1997; Sjostrom et al. 2001; Debanne et al. 1998; Bi and Poo 2001) neurons, is usually termed Spike Timing Dependent Plasticity (STDP).

The role of STDP has been investigated both in supervised learning framework (Legenstein et al. 2005), in unsupervised framework in which repeating patterns are detected by downstream neurons (Masquelier et al. 2009, Gilson et al. 2011), cortical development (Song and Abbot 2001), generation of sequences (Fiete et al. 2010; Verduzco- Flores et al. 2011) and polychronous activity (Izhikevich 2006), and in an associative memory framework with binary units (Scarpetta et al. 2011a, b). However, this is the first time that this learning rule has been used to make a IF network to work as associative memory for phase-coded patterns of spike, each of which becomes a dynamic attractor of the network. Notably, in a phase coded pattern not only the order of activation matters, but the precise spike timing intervals between units.

We therefore present a possibility to build a circuit with stable phase relationships between the spikes of a population of IF neurons, in a robust way with respect to noise and changes of frequency. The first important result of the paper is the measurement of the storage capacity of the model, i.e. the maximum number of distinct spatiotemporal patterns that can be stored and selectively retrieved, since it has never been computed in a spiking model for spatiotemporal patterns.

Several classic papers (see Scarpetta et al. 2001, and references therein) have focused on storage capacity of binary model with static binary patterns (Hopfield 1982), and much efforts have been done to use more biophysical models and patterns (Gerstner et al. 1996, 1993; Hopfield 1995; De Almeida et al. 2007; Amit and Treves 1989; Battaglia and Treves 1998; Anishchenko and Treves 2006; Borisyuk and Hoppensteadt 1998; Leibold and Kempter 2006; Memmesheimer and Timme 2006; Olmi et al. 2010; Scarpetta et al. 2002, 2011b), but, up to our knowledge, without any calculation of the storage capacity of spatiotemporal patterns in IF spiking models. Notably, by introducing an order-parameter which measures the overlap between phase coded spike trains, we are able quantitatively measure of the overlap between the stored pattern and the replay activity, and to compute the storage capacity as a function of the model parameters.

Another important result is the study of the different regimes observed by changing the excitability parameters of the network. In particular, we find that near the region of the parameter space where the network tends to become unresponsive and silent there is a regime in which the network responds selectively to cue presentation with a short transient replay of the phase-coded pattern. Differently, in the region of higher excitability, the patterns are replayed persistently and selectively, and eventually with more then one spike per cycle.

The paper is organized as follows: Section 2 introduces the Leaky-Integrate-and-Fire (IF) neuronal model; Section 3 describes the STDP learning rule used to design the connections; in Section 4 we study the emergence of collective dynamics and introduce an order parameter to measure the overlap between the collective dynamics and the stored phase coded patterns; Section 5 reports on the storage capacity of the network, i.e. the maximum number of patterns that can be stored and selectively retrieved in the network; the parameter space and the different working regions are also investigated in Section 5; in Section 6 we study the robustness of the retrieval dynamics wrt noise and heterogeneity; Section 7 reports on the implication of this model in the framework of oscillatory interference model of path-integration; summary and discussion are outlined in Section 8.

## The model

We consider a recurrent neural network with *N*(*N* − 1) possible connections *J*_*ij*_, where *N* is the number of neural units. The connections *J*_*ij*_ are designed during the learning mode, when the connections change their efficacy according to a learning rule inspired to the STDP. After the learning stage, the connections values are frozen, and the collective dynamics is studied. This distinction in two stages, plastic connection in the learning mode and frozen connections in the dynamics mode, is a useful framework to simplify the analysis. It also finds some neurophysiological motivations in the effects of neuromodulators, such as dopamine and acetylcholine (Hasselmo 1993, 1999), which regulate excitability and plasticity.

The single neuron model is a Leaky Integrate-and-Fire (IF) (Gerstner and Kistler 2002). This simple choice, with few parameters for each neuron, is suitable to study the emergence of collective dynamics and the diverse regimes of the dynamics, instead of focusing on the complexity of the neuronal internal structure. We use the Spike Response Model (SRM) formulation (Gerstner and Kistler 2002; Gerstner et al. 1993) of the IF model, which allows us to use an event-driven programming and makes the numerical simulations faster with respect to a differential equation formulation.

In this picture, the postsynaptic membrane potential is given by:

1 $$ h_i(t)=\sum\limits_{j} J_{ij} \sum\limits_{ {\hat t}_j > {\hat t}_i} \epsilon\left(t-{\hat t}_j\right), \label{IF} $$

where *J*_*ij*_ are the synaptic connections, *ε*(*t*) describes the response kernel to incoming spikes on neuron *i*, and the sum over ${\hat t}_j$ runs over all presynaptic firing times following the last spike of neuron *i*. Namely, each presynaptic spike *j*, with arrival time $\hat{t}_j$, is supposed to add to the membrane potential a postsynaptic potential of the form $J_{ij} \epsilon(t-{\hat t}_j)$, where

2 $$\begin{array}{lll} \epsilon\left(t-{\hat t}_j\right)\nonumber\\ \quad = K \left[\! \exp\left(-\frac{t-{\hat t}_j}{\tau_m}\right) - \exp\left(-\frac{t-{\hat t}_j}{\tau_s}\right) \!\right] \Theta\left(t-{\hat t}_j\right) \label{tre} \end{array}$$

where *τ*_*m*_ is the membrane time constant (here 10 ms), *τ*_*s*_ is the synapse time constant (here 5 ms), *Θ* is the Heaviside step function, and K is a multiplicative constant chosen so that the maximum value of the kernel is 1. The sign of the synaptic connection *J*_*ij*_ sets the sign of the postsynaptic potential’s change, so there’s inhibition for negative *J*_*ij*_ and excitation for positive *J*_*ij*_.

When the membrane potential *h*_*i*_(*t*) exceeds the spiking threshold $\theta_{\rm th}^i$, a spike is scheduled, and the membrane potential is reset to the resting value zero. We use the same threshold *θ*_th_ for all the units, except in Section 6 where different values $\theta_{\rm th}^i$ are used and the robustness w.r.t. the heterogeneity is studied. Clearly the spiking threshold *θ*_th_ of the neurons is related to the excitability of the network, an increase of the value of *θ*_th_ is also equivalent to a decrease of K, the size of the unitary postsynaptic potential, or, equivalently to a global decrease in the scaling factor of synaptic connections *J*_*ij*_.

Numerical simulations of this dynamics are performed for a network with *P* stored patterns, where connections *J*_*ij*_ are determined via a learning rule described in the next paragraph. We found that a few number of spikes, given a in proper time order, are able to selectively induce the emergence of a persistent collective spatiotemporal pattern, which replays one of the stored pattern (see Section 4).

## Designing the connections of the network

In a learning model previously introduced in Scarpetta et al. (2001, 2002) and Yoshioka et al. (2007), the average change in the connection *J*_*ij*_, occurring in the time interval [ − *t*_learn_,0] due to periodic spike trains of period T, with *t*_learn_ > > *T*, was formulated as follows:

3 $$ \delta J_{ij} = \frac{T}{t_{\rm learn}} \int\limits_{-t_{\rm learn}}^{0}d t \int\limits_{-t_{\rm learn}}^{0}d t^\prime \, x_i(t) A(t-t^\prime) x_j(t^\prime) \label{lr} $$

where *T*/*t*_learn_ is a normalization factor, *x*_*j*_(*t*) is the activity of the pre-synaptic neuron at time t, and *x*_*i*_(*t*) the activity of the post-synaptic one. It means that the probability for unit *i* to have a spike in the interval (*t*,*t* + *Δt*) is proportional to *x*_*i*_(*t*)*Δt* in the limit *Δt*→0. The learning window A(*τ* = *t* − *t*′) is the measure of the strength of synaptic change when a time delay *τ* occurs between pre and post-synaptic activity. To model the experimental results of STDP in hippocampal neurons, the learning window *A*(*τ*) should be an asymmetric function of *τ*, mainly positive (LTP) for *τ* > 0 and mainly negative (LTD) for *τ* < 0.

Equation (3) holds for activity pattern *x*(*t*) which represents instantaneous firing rate, and is suitable to use in analog rate models (Scarpetta et al. 2001, 2002, 2008; Yoshioka et al. 2007; Scarpetta and Marinaro 2005) and spin network models (Scarpetta et al. 2010, 2011b). Differently, here, being interested in spiking neurons, the patterns to be stored are defined as precise periodic sequence of spikes, i.e. spike-phase coded patterns. Namely, activity of the neuron *j* is a spike train at times $t^\mu_j$,

4 $$ x_j^\mu(t)=\sum\limits_n \delta(t- (t^\mu_j+ n T^\mu)), \label{spiketr1} $$

where $t^\mu_j+n T^\mu$ is the set of spikes times of unit j in the pattern *μ* with period *T* ^*μ*^, and frequency *ν* ^*μ*^ = 1/*T* ^*μ*^. Therefore, following Eq. (3), the change in the connections *J*_*ij*_ due to the learning of the pattern *μ* when the time duration of the learning process *t*_learn_ is longer then a single period *T* ^*μ*^, is simply given by

5 $$ J_{ij}^\mu=\sum\limits_{n=-\infty}^{\infty} A(t^\mu_j-t^\mu_i+ n T^\mu). \label{lr2} $$

**Fig. 1:**
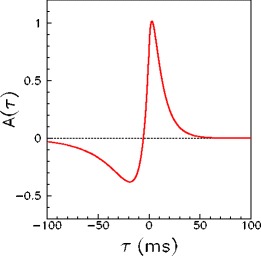
(**a**) Plot of the learning window *A*(*τ*) used in the learning rule (see Eqs. (3), (5) and (6)) to model STDP effects. The parameters of the function *A*(*τ*) (Eq. (6)) are determined by fitting the experimental data reported in Bi and Poo (1998)

The window *A*(*τ*), shown in Fig. 1, is given by

6 $$ A(\tau) = \left \{ \begin{array}{ll} a_p e^{-\tau/T_p} - a_{\tiny D} e^{-\eta \tau/T_p}& \quad \textrm{for}\quad \tau>0 \\ a_p e^{\eta\tau/T_{\tiny D}} - a_{\tiny D} e^{\tau/T_{\tiny D}} & \quad \textrm{for}\quad \tau<0, \end{array}\right. \label{At} $$

with the same parameters used in Abarbanel et al. (2002) to fit the experimental data of Bi and Poo (1998), namely *a*_*p*_ = *γ*[1/*T*_*p*_ + $\eta/T_D]^{-1}$, *a*_*D*_ = *γ*[*η*/*T*_*p*_ + $1/T_D]^{-1}$, with *T*_*p*_ = 10.2 ms, *T*_*D*_ = 28.6 ms, *η* = 4, *γ* = 0.42. This function satisfies the balance condition $\int_{-\infty}^\infty A(\tau) d\tau =0$. Notably, when *A*(*τ*) is used in Eq. (5) to learn phase-coded patterns with uniformly distributed phases, then the property $\int A(\tau) dt = 0$ assures that in the connection matrix the summed excitation $(1/N)\sum_{i, J_{ij}>0} J_ {ij}$ and the summed inhibition $(1/N)\sum_{i, J_{ij}<0} J_ {ij}$ are equal in the thermodynamic limit, and therefore it assures a balance between excitation and inhibition.

Writing Eqs. (3–5), implicitly we have assumed that, with periodic phase-coded spike trains used to induce plasticity, the effects of all separate spike pairs sum linearly, each weighted by the same STDP window reported in Fig. 1. Timing-dependent learning curves as the one reported in Fig. 1 are indeed typically measured by giving an order of 100 pairs of spikes repeatly, with fixed phase relationship, and fixed frequency in a proper range.

However, in different situations, for instance if the frequency is too low or to high (Sjostrom et al. 2001), or in case of few spike pairs (Wittenberg and Wang 2006), the timing dependence of plasticity is not well described by the bidirectional window used here, and a more detailed model is needed to account for integration of spike pairs when arbitrary trains are used (see Shouval et al. 2010; Graupner and Brunel 2010, and references therein).

The spikes patterns used in this work are periodic spatiotemporal sequences, made up of one spike per cycle and each of which has a phase $\phi^\mu_j$ randomly chosen from a uniform distribution in [0,2*π*). In each pattern, information is coded in the precise time delay between spikes of unit *i* and unit *j*, which corresponds to a precise phase relationship among units *i* and *j*. A spatiotemporal pattern represented in this way is often called phase coded pattern. Pattern’s information is coded in the spiking phases which, in turn, shape the synaptic connectivity responsible of the emerging dynamics and the memory formation.

The set of timing of spikes of unit *j* can be defined as $t^\mu_j + n T^\mu =(\phi^\mu_j)/(2\pi \nu^\mu) + n/\nu^\mu$, where *ν* ^*μ*^ is the oscillation frequency of the neurons. Thus, each pattern *μ* is represented through the frequency *ν* ^*μ*^ and the specific phases of spike $\phi^\mu_j$ of the neurons *j* = 1,..,*N*. The change in the connection *J*_*ij*_ provided by the learning of pattern *μ* is given by

7 $$\begin{array}{rll} J_{ij}^\mu&=&\displaystyle\sum\limits_{n} A(t^\mu_j-t^\mu_i+n T^\mu)\nonumber\\ &=& \sum\limits_n A\left(\frac{\phi^\mu_j}{ 2\pi \nu^\mu}-\frac{\phi^\mu_i}{2\pi\nu^\mu} +n/\nu^\mu \right). \label{conn} \end{array}$$

When multiple phase coded patterns are stored, the learned connections are simply the sum of the contributions from individual patterns, namely

8 $$ J_{ij}=\sum\limits_{\mu=1}^P J^{\mu}_{ij}. \label{connP} $$

Note that ring-like topology with strong unidirectional connections is formed only in the case P=1, when a single pattern is stored. When multiple patterns are stored in the same connectivity, with phases of one pattern uncorrelated with the others, bidirectional connections are possible, and the more the stored patterns, the less the ring-like is the connectivity. Even in the cases when the connectivity is not ring-like the network is still able to retrieve each of the P stored patterns in a proper range of threshold values (see storage capacity in Section 5).

## Emerging of collective patterns in the neural dynamics of the network

We study a recurrent network with *N* leaky Integrate and Fire units, with connections fixed to the values calculated in Eqs. (7) and (8) for different values of P. The results show that, within a well specified range of parameters, our IF network is able to work as an associative memory for spike-phase patterns.

In order to check if the network is able to retrieve selectively each of the stored patterns, we give an initial signal, made up of *M* ≪ *N* spikes, taken from the stored pattern *μ*, and we check if this initial short cue is able to selectively trigger a collective sustained activity that is the replay of the same stored pattern *μ*, i.e. checking if the sustained activity has spikes aligned to the phases $\phi_i^\mu$ of pattern *μ*.

**Fig. 2:**
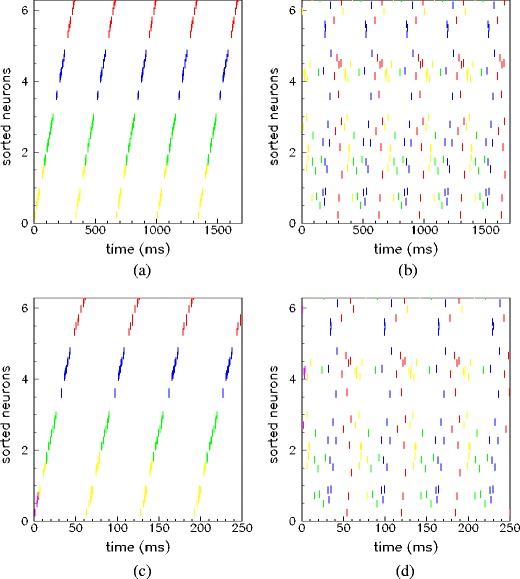
Examples of selective successful retrieval ((**c**), (**d**)) of two stored patterns ((**a**), (**b**)). The raster plot of 50 units (randomly chosen) are shown sorted on the *vertical axis* according to increasing values of phase $\phi_i^1$ of the first stored pattern *μ* = 1. The network has *N* = 3000 IF neurons, *Θ*_th_ = 70 and connections given by Eqs. (7) and (8) with *P* = 5 stored patterns at *ν* ^*μ*^ = 3 Hz. Two of the stored patterns used during the learning mode are shown in (**a**), (**b**). The dynamics emerging after a short train of *M* = *N*/10 spikes with phases similar to the pattern shown in (**a**) and (**b**), is shown in (**c**) and (**d**) respectively. The dynamics of the network, after a transient, is periodic of period *T*. The spikes which belong to the trigger are shown in pink in (**c**) and (**d**), the other different colors represent the value of $t_i/T\!\mod 4$, where *t*_*i*_ is the time of the spike of the unit i during the emerging spontaneous dynamics. Figure (**c**) shows that when the network dynamic is stimulated by a partial cue of pattern *μ* = 1, the neurons oscillate with phase alignments resembling pattern *μ* = 1, but at different frequency. Otherwise, in (**d**), when the partial cue is taken from pattern *μ* = 2, the neurons phase relationships, even if periodic, are uncorrelated with pattern *μ* = 1, and recall the phase of pattern *μ* = 2

An example of successful selective retrieval process is shown in Fig. 2 where, depending on the partial cue presented to the network, a different collective activity emerges with the phases of the firing neurons which resemble one or another of the stored patterns.

In this work the cue is a stimulation with *M* spikes, with *M* = *N*/10, at times $t^\mu_i= T_{\rm stim} \phi_i^\mu$, 0 < *i* < *M*, with *T*_stim_ = 50 ms. In the example shown in Fig. 2 the short stimulation (which lasts less then 5 ms, shown in pink in all the figures) has the effect to selectively trigger the sustained replay of pattern *μ*.

Note that the retrieval dynamics has the same phase relationship among units than the stored pattern, but the replay may happen on a time scale different from the scale used to store the pattern, and the collective spontaneous dynamics is a time compressed (or dilated) replay of the stored pattern. Indeed, the period of the collective periodic pattern which emerges during retrieval stage may be different then the period of the periodic pattern used in the learning stage. In the example of Fig. 2 the time scale of the retrieval dynamics (Fig. 2(c), (d)) is faster then the time scale used to learn the patterns (Fig. 2(a), (b)). In the following we will study the factors affecting the time scale during retrieval, given the time scale of the pattern used during learning.

**Fig. 3:**
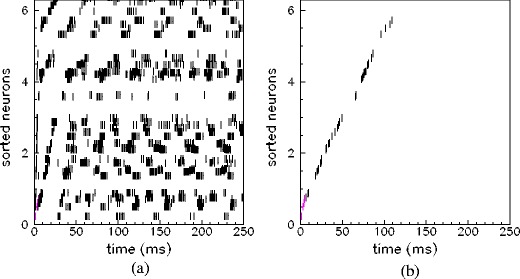
Example of neural response in two case of failure of retrieval. A spurious state emerge in (**a**), while a short transient response emerges in the case shown in (**b**). *N* = 3000 and *ν* ^*μ*^ = 3 Hz as in previous picture, while the values *θ*_th_ and *P* are *θ*_th_ = 10, *P* = 5 in (**a**) and *θ*_th_ = 95, *P* = 5 in (**b**). The dynamics emerging after a short train of *M* = *N*/10 spikes with phases equal to the stored pattern (pattern shown in Fig. 2(a)), is not a self-sustained retrieval of the pattern. For clarity, the raster plot of only 50 (randomly chosen) units are shown, sorted according to increasing value of phase *φ*_*i*_^1^ of the stored pattern

Clearly, regions of the parameter space in which the network is unable to retrieve selectively the patterns also exist. In these regions the retrieval dynamics may correspond to a mixture of patterns or a spurious state, i.e. a state which is not correlated with any of the stored patterns because the number of stored patterns exceeded the storage capacity of the network. As discussed below, the storage capacity, defined as the maximum number of encoded and successfully retrieved patterns, depends on the frequency used during the learning stage (which affects connectivity), and on the spiking threshold of the units (which affects excitability and network dynamics). Example of failure are shown in Fig. 3. In Fig. 3(b) the network has too low excitability and the response is not persistent, while in Fig. 3(a) the emerging dynamics is not correlated with any of the stored patterns.

To measure quantitatively the success of the retrieval, in analogy with the Hopfield model, we introduce an order parameter, which estimates the overlap between the network collective activity during the spontaneous dynamics and the stored phase-coded pattern. This quantity is 1 when the phases *φ*_*j*_ of neurons *j* coincides with the stored phases $\phi_j^\mu$, and is close to zero when the phases are uncorrelated with the stored ones. Therefore, we consider the following dot product |*m* ^*μ*^(*t*)| = < *ξ*(*t*)|*ξ* ^*μ*^ > where *ξ* ^*μ*^ is the vector having components $ e^{i \phi_j^\mu}$, namely:

9 $$ |m^\mu(t)|= \left|\frac{1}{N}\sum\limits_{\stackrel{j=1,\ldots,N}{\scriptscriptstyle t-T^*<t_j^*<t}} e^{-i 2 \pi t_j^*/T^*} e^{i \phi_j^\mu}\right| \label{nn} $$

where $t_j^*$ is the spike timing of neuron *j* during the spontaneous dynamics, and *T* ^*^ is an estimation of the period of the collective spontaneous periodic dynamics. The overlap in Eq. (9) is equal to 1 when the phase-coded pattern is perfectly retrieved (i.e. same sequence and phase relationships among spikes, even though on a different time scale), while is of order $\simeq 1/\sqrt{N}$ when phases of spikes are uncorrelated to the stored phases. The order parameter |*m* ^*μ*^| allow us to measure the network storage capacity in the space of parameters *θ*_th_ and *ν* ^*μ*^.

Note that the value of *m* ^*μ*^(*t*) between two periodic spike trains measures the similarity in the sequence of spiking neurons and in the phase lag between the spikes, being invariant by a simple change in time scale. This is a suitable choice especially when the replay of a spatio temporal pattern has to be detected independently from the compression of the time scale. Note that if we have a spike train that is not periodic, we cannot define the period, however we can define the order parameter Eq. (9) looking at the time-window *T* ^*^ which maximize the order parameter. This can be useful in the case when one looks for a short replay hidden in a not-periodic spike train, such in many experimental situations.

The value of *m* ^*μ*^(*t*) after a transient converges to a stable value which is close to one when pattern *μ* is retrieved (for example in Fig. 2(c) at large times *m* ^*μ* = 1^ = 1, and *m* ^*μ* = 2^ = 0.01) while *m* ^*μ*^(*t*) is of order $\simeq 1/\sqrt{N}$ for all *μ* in the case of failure of retrieval. Two further cases of failure can occur: in Fig. 3(a) *m* ^*μ*^(*t*) after the transient has values in the range 0.01–0.02 for all *μ* because the emerging dynamics is a spurious state not correlated with any of the stored phase-patterns, while *m* ^*μ*^(*t*) is zero in Fig. 3(b) since the network becomes silent.

In the following, the storage capacity of the model is analyzed considering the maximum number of patterns that the network is able to store and selectively recall. In particular we investigate the role of two model parameters: the frequency of the stored patterns *ν*_*μ*_, and the spiking threshold *θ*_th_ affecting the excitability of the network.

## Storage capacity

Numerical simulations of the IF network with *N* = 3000 neurons were performed by systematically changing the value of the spiking threshold *θ*_th_, the connections *J*_*ij*_, and for different number of patterns P and frequency *ν*_*μ*_. Here we propose results for a unique value of the spiking threshold *θ*_th_ for all neurons, however the behavior is also robust with respect to a variability in the threshold values among neurons, as reported in the next section.

Network storage capacity is defined as *α*_*c*_ = *P*_max_/*N*, where N is the number of neurons and *P*_max_ is the maximum number of patterns that can be stored and successfully retrieved with an overlap |*m* ^*μ*^| larger than a certain value, which measures the degree of similarity. Given that in our simulations the overlap |*m* ^*μ*^(*t*)| at large times has mostly two possible values, close to one (success) or close to $1/\sqrt N$ (failure), we fixed the desired similarity value to 0.5, since the whole storage capacity analysis is very robust with respect to this parameter (since the transition between low values and high values of |*m* ^*μ*^(*t*)| as a function of P is sharp).

Patterns with random phases were extracted and used to define the network connections *J*_*ij*_ with the rule Eq. (8). After the stimulation with a short train of *M* = *N*/10 spikes taken at times *t*_*i*_ from the first pattern, the dynamics is simulated and the overlap defined in Eq. (9) with *μ* = 1 is evaluated at large times. If the overlap |*m* ^*μ* = 1^(*t*)|, averaged over 50 runs, is greater than 0.5 at time $t>\bar t$ (where $\bar t= 600$ ms for all the simulations), then we consider the retrieval successful for that pattern. The maximum value of P, for which the network is able to successfully replay each of the stored patterns, defines the storage capacity of the network.

**Fig. 4:**
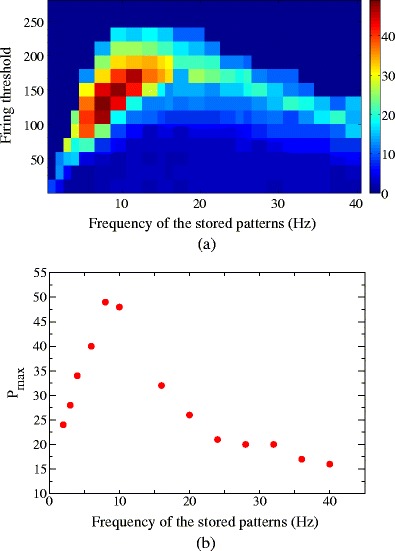
(**a**) Storage capacity in a network of *N* = 3000 units, as a function of the spiking threshold *θ*_th_ and oscillation frequency *ν* ^*μ*^ of stored patterns. The maximum number of patterns successful retrievable *P*_max_ is shown in color-coded legend, the value grows from *P*_max_ = 0 (*dark blue*) to *P*_max_ = 50 (*strong red*). (**b**) The storage capacity *P*_max_ as a function of the frequency of stored patterns, once fixed the threshold *θ*_th_ to the optimal value for each frequency

The storage capacity as a function of the spiking threshold *θ*_th_ and storing frequency *ν* ^*μ*^ is reported in Fig. 4(a), where *P*_max_ is shown in a color-coded legend. The largest capacity is achieved when the frequency of the stored patterns during learning is *ν* ^*μ*^ ≃ 8 Hz and the spiking threshold of the units during retrieval is *θ*_th_ ≃ 130, which provides a capacity *α*_max_ = *P*_max_/*N* = 0.016.

In Fig. 4(b) we show the storage capacity *P*_max_ as a function of the frequency *ν*_*μ*_ once fixed the value of the threshold *θ*_th_ to the optimal value, corresponding to highest capacity for each frequency.

The optimal storing frequencies and threshold values depend on the time constants of the model, such as the *τ*_*s*_,*τ*_*m*_ of the IF units and the temporal shape of the learning kernel *A*(*τ*), whereas different shapes of *A*(*τ*) may subserve to different storing frequency ranges. In this work *τ*_*s*_,*τ*_*m*_ and *A*(*τ*) are set to the values described in Section 2, and the emergent collective dynamics is studied as a function of the other network parameters. Indeed, Fig. 4(b) shows that for the learning kernel *A*(*τ*) used here, there is peak in the storage capacity around 8 Hz, in the range 2–20 Hz. Figure 4(a) also proves that, for each stored frequency, a large interval of spiking threshold values *θ*_th_ exists for which the network is still able to work properly as associative memory for phase-coded patterns.

**Fig. 5:**
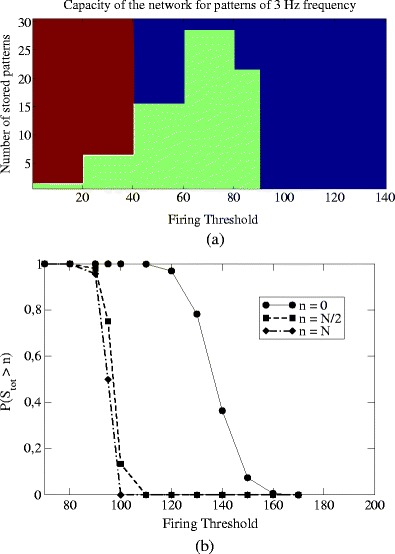
(**a**) Storage capacity at *ν* ^*μ*^ = 3 Hz: the region of successful retrieval as a function of spiking threshold and number of patterns is marked in *green*. The region with persistent activity not correlated with any of the stored pattern is marked in *red* (spurious states), and the region in which the network responds with only a short transient and then becomes silent is marked in *blue* (see examples in Fig. 3). (**b**) The probability that the size *S*_tot_ of the network response, measured as the number of the spikes that follow the cue stimulation, is larger than *n*, with *n* = 0,*N*/2,*N*, is shown as a function of spiking threshold *θ*_th_, in a network with *ν* ^*μ*^ = 3 Hz and *P* = 1. As always in this paper the number of units is *N* = 3000. The figure shows that near $\theta_{\rm th}^{\rm crit} \simeq 90$ there’s a transition from a region of persistent replay to a region of silence

The associative memory properties as a function of the spiking threshold are reported in Fig. 5, when the oscillation frequency of the patterns stored during learning is *ν* ^*μ*^ = 3 Hz. The region marked in green in Fig. 5 corresponds to cases in which the retrieval is successful and the cue is able to selectively activate the self-sustained replay of the stored pattern (with an order parameter *m* ^*μ*^ larger than 0.5). When spiking threshold changes in the range 10 < *θ*_th_ < 90 the storage capacity changes between *P*_max_/*N* = 1/3000 and *P*_max_/*N* = 29/3000.

Outside the green region the number of patterns exceeds the storage capacity and the retrieval fails. There are two possible reasons for this behavior. At low threshold, when the number *P* of patterns exceeds the storage capacity *P*_max_, the network responds with a self-sustained activity that is not correlated with any of the stored patterns, i.e. a spurious state. In this regime, marked with red color in Fig. 5(a), the order parameter *m* ^*μ*^(*t*) is of order $1/\sqrt N$ for all the stored patterns (see also raster plot in Fig.  3(a)). On the contrary, in the high *θ*_th_ regime, the network tends to become silent and unresponsive. Indeed, in the region marked with blue color, the network responds to the initial cue stimulation with a short transient and then became silent. In this case the value of *m* ^*μ*^(*t*) is zero because there is no self-sustained activity at time $t>\bar t$, meaning that the stored attractors become unstable when *θ*_th_ is too high (see raster plot in Fig. 3(b)).

For values of the threshold greater than $\theta_{\rm th}^{\rm crit}$, independently from P, the network activity is never persistent, as reported in Fig. 5(a) where $\theta_{\rm th}^{\rm crit}=90$.

At thresholds close to this critical value the network responds with a transient activity that is a short replay of the stored pattern, but not a persistent replay. The size *S*_tot_ of the network response, measured as the number of spikes that follow the cue stimulation, is reported in Fig. 5(b) as a function of *θ*_th_, for a network with *ν* ^*μ*^ = 3 Hz, *N* = 3000 and *P* = 1.

**Fig. 6:**
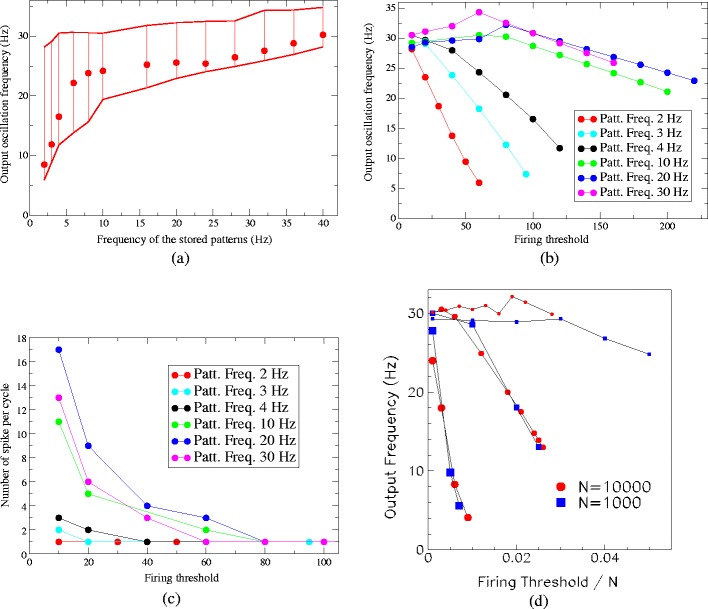
(**a**) Frequency of the collective dynamics during replay as a function of the frequency of stored patterns in the network with *N* = 3000 units. *Dots* refer to replay frequency observed at optimal spiking threshold. The *bars* refers to the range of frequency available through changes in spiking threshold. (**b**) Frequencies of the collective dynamics during replay as a function of the spiking threshold and for different stored frequencies (see colors legend). Pattern is replayed on a time scale which becomes faster if we decrease threshold *θ*_th_ for the most of the frequencies *ν* ^*μ*^. The dependence is much stronger for *ν* ^*μ*^ ≤ 4 Hz. *N* = 3000. (**c**) The number of spikes per cycle as a function of the spiking threshold *θ*_th_ in networks with different frequency *ν* ^*μ*^ of stored pattern. (**d**) Frequency of the dynamics during replay as a function of ratio between spiking threshold and network size N. *Red dots* are results for a network with *N* = 10000 units, while *blue squares* are results for a network with *N* = 1000 units. Size of the symbols refers to the stored frequency, small symbols (*on the top of the picture*) correspond to stored frequency *ν* ^*μ*^ = 10 Hz, medium size symbols correspond to stored frequency *ν* ^*μ*^ = 3 Hz, and large symbols (*bottom*) correspond to stored frequency *ν* ^*μ*^ = 1 Hz

In the following we investigate the replay activity in the region with successful retrieval. We focused on the dependence of the frequency of collective oscillations during replay on the model parameters. Figure 6(a) shows the collective frequency of replay as a function of the frequency *ν* ^*μ*^ of the patterns stored in the learning stage with *N* = 3000. The red dots in the figure refer to the frequencies of oscillations observed during retrieval at the optimal spiking threshold (where the maximum storage capacity occurs), while the bar indicates the available range of frequencies of replay, accessible through a change in the spiking threshold. Important to note is that, in most of the cases, the frequency of the stored pattern and the collective replay frequency do not coincide, since the pattern is replayed compressed (or dilated) in time, on a time scale dependent on the network parameters. We observe that for the chosen parameters *τ*_*m*_, *τ*_*s*_ of the network, and the given shape of *A*(*τ*), the replay occurs on a compressed time scale for all stored patterns of frequency lower then 25 Hz, while the two time scale coincide when *ν* ^*μ*^ ≃ 25 Hz. The dependence of the frequency of the collective oscillations on the spiking threshold is shown in Fig. 6(b). This dependence is weak for stored frequencies higher than 10 Hz. Besides, for low stored frequencies (1–4 Hz) the frequency of the replay is very sensitive to the threshold value, changing from 6 Hz at high spiking threshold to 30 Hz at low threshold.

We also investigate the frequency’s dependence on network size *N*. In Fig. 6(d) red dots are results for a network with *N* = 10000 units, while blue squares are results for a network with *N* = 1000 units. If what counts is only the time lag between the single units consecutive in the sequence, then one expects that result with 1 Hz stored at *N* = 10000 would be similar to 10 Hz stored at *N* = 1000, and this is not the case. We see that when we use a storage frequency equal to 10 Hz (which corresponds to different time lag between cells depending on N), then the oscillation frequency during replay is around 30 Hz in both networks (both *N* = 1000, and *N* = 10000), while, on the other hand, if we have a storage frequency equal to 1 Hz the oscillation frequency during replay may span a large range (5 Hz–25 Hz) in both networks. Figure 6(d) also shows that frequency of replay depends on the ratio between spiking threshold and network size N, and that the high sensitivity on spiking threshold value holds, when stored frequency is low (1–4 Hz), also at different values of the network size.

This open the possibility to govern the oscillation frequency of the collective replay activity via neuromodulators which change the excitability and therefore the spiking threshold of the neurons. Since in our model (see Eqs. (1) and (2)) a change of the threshold is equivalent to a change in the scale factor of all synaptic connections, a similar effect might be achieved also by simply driving the cells more due to increased synaptic input. Importantly, the sensitivity of collective oscillation frequency on spiking threshold is not a sensitivity of the single unit but of the collective behavior, since, as discussed in Section 6, if we change the spiking threshold of few units the collective rhythm is still unique for the whole population. The replay frequency depends on the average threshold among units, but all the units have the same oscillation frequency during replay.

**Fig. 7:**
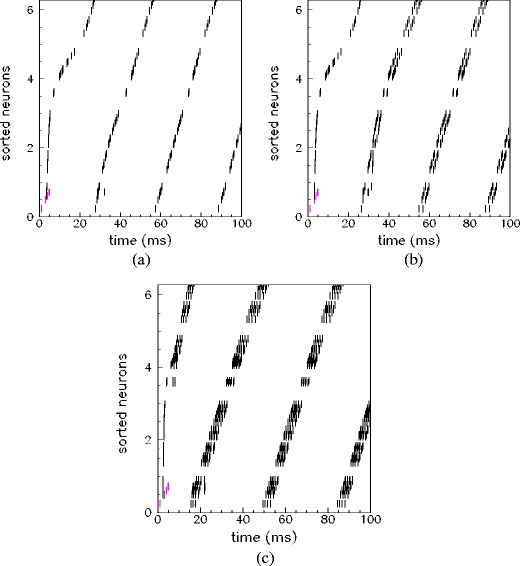
Modulation of spiking threshold changes the number of spikes per cycle, keeping preserved the phase relationship among units. Recall of the pattern *μ* = 1 for networks of *N* = 3000 units, *ν* ^*μ*^ = 20 Hz and different values of spiking threshold *θ*_th_ = 80,65,40 is shown respectively in (**a**)–(**c**). Depending on the value of the spiking threshold *θ*_th_, the phase-coded pattern is replayed with a different number of spikes per cycle. Spike of the cue stimulation are shown in *pink*, while the response of the network in *black*. For clarity, the raster plot of only 50 (randomly chosen) units are shown, sorted according to increasing value of phase *φ*_*i*_^1^ of the stored pattern

Moreover, for networks with *ν* ^*μ*^ ≥ 10 Hz, whose replay frequency does not considerably change with spiking threshold, the replay dynamics is still affected by the spiking threshold. Indeed, in this case, the number of spikes per cycle increases with lowering of the spiking threshold. An example is reported in Fig. 7. The raster plots show the same pattern replayed in three networks having different values of the spiking threshold *θ*_th_: a burst of activity takes place within each cycle, with phases aligned with the pattern, with a number of spikes per cycle dependent on the value of *θ*_th_. This behavior is summarized in Fig. 6(c) where the number of spikes per cycle is reported as a function of spiking threshold, at different values of stored frequencies. Therefore, by lowering the spiking threshold the replay activity occurs with more than one spike per cycle, or on a faster time scale (see Fig. 6(b), (c)).

The behavior of the output oscillation frequency suggests that a parameters region exists where the network always responds with one spike per cycle. In this region an increase of the excitability produces a growth of the frequency of oscillation up to a plateau value. Differently, for higher excitability the frequency does not increase while the number of spikes per cycle grows. This means that the different frequencies, in addition to the information coded in the phase relationship, can code other information in relationship with the level of spiking threshold: at high frequency the threshold changes the number of spikes per cycle, while at low frequency the threshold changes the frequency of the collective oscillations during the replay.

This open the possibility to have a coding scheme in which, while the phases encode pattern’s information, a change in frequency or a change in rate in each cycle represents the strength and saliency of the retrieval or it may encode another variable (Lengyel and Dayan 2007). The recall of the same phase-coded pattern with a different number of spikes per cycle is particularly interesting at the light of recent observations of Huxter et al. (2003) in hippocampal place cells, showing the occurrence of the same phases with different rates. The authors prove that the phase of firing and firing rate are dissociable and can represent two independent variables, e.g. the animal location within the place field and its speed of movement through the field.

Notably, the recall of the same phase coded pattern with different frequencies of oscillation is also relevant and accords well with the need to have stable precise phase relationship among cells with frequency of oscillation modulated by parameters such as the speed of the animal (Blair et al. 2008; Welday et al. 2011).

The value of the frequency of collective activity during the replay clearly is related not only to the threshold and the stored frequency, but also to the shape of the learning window *A*(*τ*) and on the two characteristic times of the model *τ*_*s*_, *τ*_*m*_. A systematic study of the dependence of the replay time scale on the shape of STDP and the characteristic times of the neuron model has not yet done in a spiking model, however a dependence on the asymmetry of *A*(*t*) has been analytically found in a simple model with analog neurons and a single characteristic time (Yoshioka et al. 2007).

## Effects of noise and robustness of collective oscillation frequency and phase relationships

While in the Hopfield model the patterns are static, and information is coded in a binary pattern $\bold S^\mu= S_1^\mu,\ldots,S_N^\mu$, with $S_i^\mu \in \{\pm 1\}$, here, in this study, the patterns are time dependent, and information is coded in the phase pattern $\bold{\phi}^\mu=\phi_1^\mu,...,\phi_N^\mu$ with $\phi_i^\mu \in [0,2\pi]$, where the value $\phi_i^\mu /(2\pi\nu^\mu)$ represents the time shift of the spike of unit *i* with respect to the collective rhythm, i.e the time delay among units. However, as for the Hopfield model, the patterns stored in the network are attractors of the dynamics, when *P* do not exceeds storage capacity, and the dynamics during the retrieval is robust with respect to noise. We firstly check robustness w.r.t. input noise, i.e when a Poissonian noise *η*_*i*_(*t*) is added to the postsynaptic potential *h*_*i*_(*t*) given in Eq. (1). The total postsynaptic potential of each neuron *i* is then given by

10 $$ h_i(t)=\eta_i(t)+\sum\limits_{j}J_{ij}\sum\limits_{{\hat t}_j > {\hat t}_i} \epsilon\left(t-\hat{t_j}\right) $$

where *η*_*i*_(*t*) is modelled as

11 $$ \eta_i(t)=J_{\text{noise}}\sum\limits_{\hat{t}_{\text{noise}}>\hat{t}_i} \epsilon\left(t-\hat{t}_{\text{noise}}\right). $$

The times $\hat{t}_{\text{noise}}$ are randomly extracted for each neuron *i*, and $J_{\text{noise}}$ are random strengths, extracted independently for each neuron *i* and time $\hat{t}_{\text{noise}}$. The intervals between times $\hat{t}_{\text{noise}}$ are extracted from a Poissonian distribution $P(\delta t) \propto e^{-\delta t/(N\tau_{\text{noise}})}$, while the strength $J_{\text{noise}}$ is extracted from a Gaussian distribution with mean ${\bar J}_{\text{noise}}$ and standard deviation $\sigma(J_{\text{noise}})$.

**Fig. 8:**
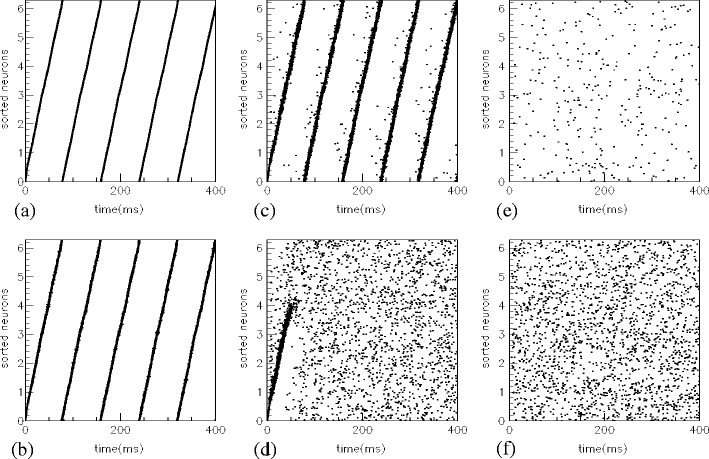
(**a**)–(**d**) Robustness wrt noise. Raster plots show that, when the pattern retrieval is triggered, network’s spikes continue to have phase alignments resembling the pattern even in presence of noise. Errors do not sum up until the system is in the basin of attraction of the phase-coded pattern, as in (**a**)–(**c**). Different levels of noise are used in (**a**)–(**d**) ($\sigma(J_{\text{noise}})=0,10,20,30$ respectively), and pattern is triggered with *M* = *N*/10 as in previous cases. Only in (**d**) the level of noise is too high and the system goes out of the basin of attraction. (**e**), (**f**) For comparison, the dynamics, when the retrieval is not triggered (*M* = 0), is shown in subplot ((**e**) and (**f**)) in presence of the same noise used in (**c**) and (**d**). Figure (**e**) shows that the noise used in (**c**) usually affects strongly the dynamics of the network, however if the collective oscillation is retrieved the system is robust wrt noise. Thresholds in all figures are *θ*_th_ = 80, *N* = 3000, and synaptic connections *J*_*ij*_ are build learning *P* = 2 phase-patterns at *ω* ^*μ*^ = 3 Hz

The network dynamics during the retrieval of a pattern in presence of noise is shown in Fig. 8 with different levels of noise ($\tau_{\text{noise}}=10$ ms, $\bar J=0 $ and $\sigma(J_{\text{noise}})=0,10,20,30$ in a, b, c and d, respectively). Results show that when the noise is not able to move the dynamics out of the basin of attraction, the errors do not sum up, and the phase relationship is preserved over time (see Fig. 8(a)–(c)). If the input noise is very high, as in the example of Fig. 8(d), the dynamics moves out of the basin of attraction.

In order to see the effects of input noise level used in Fig 8(c), (d), we report in Fig. 8(e), (f) the network dynamics when the pattern retrieval is not initiated (*M* = 0). In particular, Fig. 8(e) shows that the noise level used in Fig. 8(c) is strong enough to generate spontaneous random activity in absence of the initial triggering, but is not sufficient to destroy the attractive dynamics during a successful retrieval. As in the Hopfield model, errors do not sum up and the dynamics spontaneously goes back to the retrieved phase-coded pattern for all the perturbations that leave the system inside the basin of attraction.

**Fig. 9:**
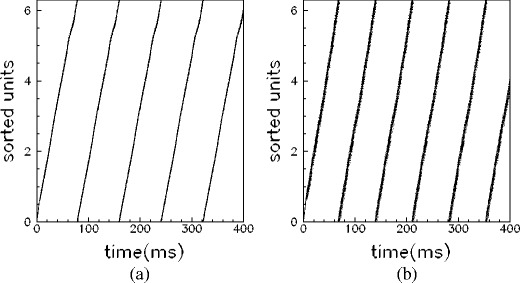
Robustness wrt heterogeneity of spiking threshold values. Raster plots show that, when the pattern retrieval is triggered, units participate to the network collective oscillation, showing all the same frequency and phase alignments resembling the pattern, even in presence of threshold values heterogeneity among units. Spiking thresholds of neuron *i* are distributed according to $\theta_{\rm th}^i=\theta_{\rm th} (1+z \zeta)$ with average *θ*_th_ = 80 and *z* = 0.2,0.5 in (**a**) and (**b**) respectively. All other parameters are as in Fig. 8(a) (*N* = 3000, *M* = *N*/10, and synaptic connections *J*_*ij*_ are build learning *P* = 2 phase-patterns at *ω* ^*μ*^ = 3 Hz)

Lastly, the robustness of retrieval w.r.t. heterogeneity of the spiking thresholds is investigated. This analysis can be carried out by using a different value $\theta_{\rm th}^i$ of spiking threshold for each neuron *i*:

12 $$ \theta_{\rm th}^i= (1 + z \zeta_i) \theta_{\rm th} $$

where *ζ*_*i*_ is a random number extracted from a uniform distribution in [ − 1,1], and *z* is the degree of heterogeneity. Even with high degree of heterogeneity, the emergence of the retrieval collective dynamics forces all neurons to have exactly the same frequency of oscillation and to keep a precise phase relationship, in a very robust manner. Figure 9(a), (b) shows the dynamics with threshold heterogeneity *z* = 0.2, 0.5 respectively, while the remaining parameters are set to the values of Fig. 8(a).

The above analysis shows that a unique collective frequency emerges, which is the frequency corresponding to the mean value of the $\theta_{\rm th}^i$. This is also evident by comparing Fig. 9(a), (b) with Fig. 8(a). At *z* = 0.5 it can be seen an additional small phase shift proportional to the value of *θ* of each neuron, but collective activity is preserved. Clearly if *z* is too high and threshold values are distributed out of the region of successful retrieval the network is unable to retrieve the pattern and failure happens, as already discussed in Section 5.

## Relationship of this model with theories of path-integration

Recently, path-integration system and the hippocampal-entorinal cortex circuit have been deeply investigated (O’Keefe and Recce 1993; O’Keefe and Burgess 2005; Burgess et al. 2007; Jeewajee et al. 2008; Blair et al. 2008; Euston et al. 2007; Geisler et al. 2007; McNaughton et al. 2006), showing that place cells and grid cells form a map in which precise phase relationship among units play a central role to generate the spatial tuning. A number of models of the spatial firing properties of place and grid cells were offered. Generally two main categories are distinguished: models which focus on continuous attractor mechanisms and models which use interference between oscillators at dynamically modulated frequencies; however, deeper computational principles may exist that unify the different cases of neural integration (Issa and Zhang 2012). In oscillatory interference models (O’Keefe and Burgess 2005; Burgess et al. 2007; Blair et al. 2008; Welday et al. 2011; Zilli and Hasselmo 2010) (see also Giocomo et al. 2011, for a review) the total synaptic input to a neuron (such as a grid cell or a place cell) is a weighted sum of the activities of n oscillatory inputs, whose oscillation frequency is modulated by the rat velocity and head direction. Grid and place cells, according to these models, derive their temporal and spatial properties simply by detecting synchrony among such velocity-modulated oscillatory inputs. The oscillatory interference theory is one possible hypothesized mechanism of path integration and it has not been conclusively accepted or rejected. It is supported by recent studies suggesting that the predicted velocity-modulated oscillators exist as theta cells (interneurons found throughout the septo-hippocampal circuit) whose inter-burst frequency shows a cosine modulation by running direction and a linear increase with running velocity (Welday et al. 2011).

Such velocity-modulated oscillatory input was hypothesized to come from single oscillatory neurons (O’Keefe and Burgess 2005), or networks such as subcortical “ring attractors” generating velocity-modulated theta oscillations (Blair et al. 2008; Zilli and Hasselmo 2010; Welday et al. 2011).

The properties of modulation and stability of frequency, and stability of multiple phase relationships, make our circuit a possible mechanism to build the velocity-modulated oscillators of the oscillatory interference theory. Indeed our circuit has a collective oscillation frequency, which depends on the frequency stored in the connectivity matrix, and that can be modulated by changing the parameters such as *θ*_th_. Each neuron in the circuit has a phase determined by its position in that network, i.e. determined by the phase $\phi_i^\mu$ of the stored phase-pattern. If the parameter *θ*_th_ is modulated by animal speed, then the collective oscillation frequency of the circuit is modulated by the animal’s speed, while the neurons preserve stable phase relationship among them.

The other persistent-firing models (Blair et al. 2008; Zilli and Hasselmo 2010; Welday et al. 2011) of the oscillators needed by the oscillatory interference theory suffer from problems related to robustness, as those encountered by the single-cell oscillatory models (Zilli et al. 2009), due to the variability in the frequency of persisting spiking (Giocomo et al. 2011). Indeed the oscillatory interference models impose strict constraints upon the dynamical properties of the velocity-modulated oscillatory inputs, which have to preserve robust velocity-modulated frequency and stable phase relationships among them on relevant time scales in a manner robust to noise (many seconds, or dozens of theta cycle periods) (Blair et al. 2008; Welinder et al. 2008; Zilli et al. 2009; Giocomo et al. 2011).

Notably, in our model, since connections *J*_*ij*_ among units in the circuit are fixed by the learning rule (Eq. (7)), dynamics is a retrieval process and neurons preserve stable precise phase relationship among units and stable frequency even in noisy conditions, at least when the dynamics is in the basin of attraction of that phase relationship.

Even in the more recent spiking models (Zilli and Hasselmo 2010; Welday et al. 2011) of the oscillatory interference principle, in which many problems related to noise are solved, the heterogeneity of parameters of the cells which participate to the ring oscillator is not taken into account.

Here we show that the circuit level interactions among units make the oscillation frequency and the phase-relationship of the system robust even with respect to heterogeneity of the spiking thresholds of the units (see Section 6).

This robustness, due to the proposed coupling which forces all the units of the circuit to have exactly the same period of oscillation and to have precisely the same phase relationships of stored pattern, may be useful in all cases of sequence coding.

Moreover, our circuit can be easily programmed to cycle in different phase orders, by storing more than one phase-pattern as attractors. The circuit is a robust phase-shuffling ring oscillator, since the network has the capability of shuffling the order in which its neurons fire, by storing a variety of different phase-patterns within the connectivity. If the oscillators predicted by the interference theory can generate more than one phase sequence, as in the model presented here, then this could provide a potential mechanism to explain the phenomenon of hippocampus remapping (Colgin et al. 2008; Wills et al. 2005). One of the more interesting discovery of place cells behavior is indeed the remapping of the place cell representation of space in response to a changes in sensory or cognitive inputs, i.e. place cells change their firing properties (place cells can appear disappear or move to other unpredictable locations). This change may be abrupt and similar to the switch from one attractor to another (Wills et al. 2005). If the place cell will fire at a specific place where its inputs become synchronized (Euston et al. 2007; Blair et al. 2008; Welday et al. 2011; Giocomo et al. 2011), by recalling a different phase-coded pattern among the ones stored in our circuit, it will change the phases of theta cells that are the inputs of the place cell, and it will change the specific “place” where the inputs become synchronized, and therefore it changes the place cell representation of the space.

Finally we note that even thou our model is not a continuous attractor, it shares many similarity with such a class of models. Our model is a circuit with many distinct attractors, one for each phase-relationship stored in the network, and the number of different attractor states is set by the maximal storage capacity studied here. Furthermore each attractor is a phase-coded pattern, replayed with a collective resonant frequency that can be modulated by changing for example the spiking threshold of the units.

Even thou during exploration the activity of place cells may be explained by the superposition of velocity-controlled oscillators inputs, the recurrent connections inside the place cells network may have anyway a relevant role.

During sleep, in absence of external input, the role of recurrent connections increases, probably due to an increase of excitability via neuromodulators or other mechanisms, and the spontaneous activity of the network show temporarily short replay of stored patterns, probably initiated simply by noise.

So the pattern activated repeatly during experience, is stored in the connectivity, and then activated during sleep when the network is near a critic point and noise is able to initiate short replay sequences.

Replay of phase-coded patterns of neural activity during sleep has been observed in hippocampus and neocortex (Schwindel and McNaughton 2011).

Notably in our model the time scale of reactivation is different from the time scale of storing, depends on the collective frequency which emerges from the connectivity, and may be accelerated or slowed down changing parameters such as spiking thresholds. Therefore, our model might be also relevant for replay in prefrontal cortex (PFC) or other cortical areas in which replay is accelerated with respect to awake activity.

In the hippocampus, spikes representing adjacent place fields occur in rapid succession within a single theta cycle during behavior. Therefore, relative to this within theta cycle rate, reactivation during sleep in hippocampus is not accelerated. However, reactivation in rat PFC is clearly compressed five to eight times relative to the waking state (Euston et al. 2007; Schwindel and McNaughton 2011). Indeed, while in hippocampus one may think that the coding sequence is the within-theta cycle, in prefrontal cortex it is clearly seen that the cross-correlation among cells during sleep replay is time compressed compared with the cross-correlation during waking state. The playback speed declines over time as does the strength of the replay, which is consistent for example with a simple increases of spiking threshold in our model.

## Discussion

We studied the temporal dynamics, including the storage and replay properties, in a network of spiking integrate and fire neurons, whose learning mechanism is based on the Spike-Timing Dependent Plasticity. The temporal patterns we consider are periodic spike-timing sequences, whose features are encoded in the relative phase shifts between neurons.

The importance of oscillations and precise temporal patterns has been pointed out in many brain structure, such as cortex (Buzsaki and Draguhn 2004), cerebellum (D’Angelo et al. 2009; D’Angelo and De Zeeuw 2009), or olfactory system (Gelperin 2006). The proposed associative memory approach, with selective replay of stored sequence, can be a method for recognize an item, by activating the same memorized pattern in response to a similar input. Another possibility is to have a way to transfer a memorized item to another area of the brain, such as for memory consolidation during sleep. During sleep, indeed, few spikes with the right phase relationship may initiate the retrieval of one of the patterns stored in the network and this reactivation may be useful for memory consolidation. The stored pattern is an attractor of the network dynamics, that is the dynamics spontaneously goes back to the retrieved phase-coded pattern for all the perturbations which leave the system within the basin of attraction. Therefore phase errors do not sum up, and the phase relationships may be transferred and kept stable over long time scales.

The time scale of the pattern during retrieval, i.e. the period of oscillation 1/*ν*, depends on (1) the time constants of the single neuron *τ*_*m*_ and *τ*_*s*_, (2) the spiking threshold *θ*_th_ of the neurons, and (3) the connectivity, through the STDP learning shape *A*(*τ*) and the time scale of the pattern during learning mode 1/*ν* ^*μ*^. Different areas of the brain may have different shape of STDP to subserve different oscillation frequencies and different functional role. Here we fix the shape of *A*(*τ*) to the one observed in hippocampal cultures (see Fig. 1 and Bi and Poo 1998) and focus on the dependence on the spiking threshold *θ*_th_ of the neurons. The spiking threshold can modulate the frequency of the collective oscillation, leaving unaffected the phase relationships among the units. This opens a possible way to govern the frequency of collective oscillation via neuromodulators, and to encode information (such as velocity of the animal) in the frequency of the oscillations, in addition to the information encoded in the phase relationships. Notably, in a particular range of frequencies, the spiking threshold does not affect the frequency of oscillation but changes the number of spike per cycle during the retrieval dynamics. This means that information can be encoded via the number of spikes per cycle, independently from the information coded in the phase relationship among units, in agreement with the observations of independent rate and phase coding in hippocampus (Huxter et al. 2003). Important to note, the phase relationships and the frequency of the collective oscillation are both robust with respect to noise and to heterogeneity of the spiking threshold of the units.

A systematic study of the retrieval capacity of the network is proposed as a function of two parameters of the model: the frequency of the input pattern and the spiking threshold. The storage capacity, evaluated as *P*_max_/*N*, is always lower than the storage capacity of the Hopfield model. However, the information content of a single pattern in our dynamical model with N units is higher than the information content of a pattern in the Hopfield model with N-units. Indeed, an Hopfield pattern is a set of N binary values while our phase-coded pattern is a set of N real number $\phi^\mu_j \in [0, 2\pi]$.

The role of STDP in the formation of sequences has been recently investigated in Verduzco-Flores et al. (2011) and Fiete et al. (2010). These studies have shown how it’s possible to form long and complex sequences, but they did not concern themselves on how it’s possible to learn and store not only the order of activation in a sequence, but the precise relative times between spikes in a closed sequence, i.e. a phase-coded pattern. In our model not only the order of activation is preserved, but also the precise phase relationship among units. The tendency to synchronization of units is avoided in our model, without need to introduce delays or adaptation, due to the balance between excitation and inhibition that is in the connectivity of large networks when the phase coded pattern with random phases is learned using the rule in Eqs. (5–8). In our rule all the connections, both positive and negative, scale with the time of presentation of patterns, keeping always a balance. Indeed, since (1) the stored phase are uniformly distributed in [0,2*π*) and (2) the learning window has the property $\int A(\tau) dt = 0$, then the connectivity matrix in Eq. (7) has the property that the summed excitation $(1/N)\sum_{i, J_{ij}>0} J_ {ij}$ and the summed inhibition $(1/N)\sum_{i, J_{ij}<0} J_ {ij}$ are equal in the thermodynamic limit (indeed they are of order unity, while their difference is of order $1/\sqrt{N}$).

Under this conditions, we investigate how multiple phase-coded patterns can be learned and selectively retrieved in the same network, as a function of time scale of patterns and network parameters.

The task of storing and recalling phase-coded memories has been also investigated in Lengyel et al. (2005b) in the framework of probabilistic inference. While we study the effects of couplings given by Eq. (5) in a network of IF neurons, the paper Lengyel et al. (2005b) studies this problem from a normative theory of autoassociative memory, in which real variable *x*_*i*_ of neuron *i* represents the neuron *i* spike timing with respect to a reference point of an ongoing field potential, and the interaction *H*(*x*_*i*_, *x*_*j*_) among units is mediated by the derivative of the synaptic plasticity rule used to store memories.

The model proposed here is a mechanism which combines oscillatory and attractor dynamics, which may be useful in many models of path-integration, as pointed out in Section 7. Our learning model offers a IF circuit able to keep robust phase-relationship among cells participating to a collective oscillation, with a modulated collective frequency, robust with respect to noise and heterogeneities. Notably the frequency of the collective oscillation in our circuit is not sensible to the single value of the threshold of each unit, but to the average value of the threshold of all units, since all units participate to a single collective oscillating pattern which is an attractor of the dynamics.

Recently there is renewed interest in reverberatory activity (Lau and Bi 2005) and in cortical spontaneous activity (Ringach 2009; Luczak and Maclean 2012) whose spatiotemporal structure seems to reflect the underlying connectivity, which in turn may be the result of the past experience stored in the connectivity.

Similarity between spontaneous and evoked cortical activities has been shown to increase with age (Berkes et al. 2011), and with repetitive presentation of the stimulus (Han et al. 2008). Interestingly, in our IF model, in order to induce spontaneous patterns of activity reminiscent of those stored during learning stage, few spikes with the right phase relationship are sufficient. It means that, even in absence of sensory stimulus, a noise with the right phase relationships may induce a pattern of activity reminiscent of a stored pattern. Therefore, by adapting the network connectivity to the phase-coded patterns observed during the learning mode, the network dynamics builds a representation of the environment and is able to replay the patterns of activity when stimulated by sense or by chance.

This mechanism of learning phase-coded patterns of activity is then a way to adapt the internal connectivity such that the network dynamics have attractors which represent the patterns of activity seen during experience of environment.
